# In vitro inhibition and reversal of *Plasmodium falciparum* cytoadherence to endothelium by monoclonal antibodies to ICAM-1 and CD36

**DOI:** 10.1186/s12936-017-1930-9

**Published:** 2017-07-05

**Authors:** Khairul M. F. Mustaffa, Janet Storm, Megan Whittaker, Tadge Szestak, Alister G. Craig

**Affiliations:** 1 Institute for Research in Molecular Medicine, Universiti Sains Malaysia, 16150 Kubang Kerian, Kelantan Malaysia; 2 Liverpool School of Tropical Medicine, Pembroke Place, Liverpool, L3 5QA UK; 3 School of Medicine, University of Liverpool, Cedar House, Ashton Street, Liverpool, L69 3GE UK

**Keywords:** *Plasmodium falciparum*, Monoclonal antibody, Adjunct therapy, Severe malaria, Cytoadherence, Reversal

## Abstract

**Background:**

Sequestration of parasitized red blood cells from the peripheral circulation during an infection with *Plasmodium falciparum* is caused by an interaction between the parasite protein PfEMP1 and receptors on the surface of host endothelial cells, known as cytoadherence. Several lines of evidence point to a link between the pathology of severe malaria and cytoadherence, therefore blocking adhesion receptors involved in this process could be a good target to inhibit pRBC sequestration and prevent disease. In a malaria endemic setting this is likely to be used as an adjunct therapy by reversing existing cytoadherence. Two well-characterized parasite lines plus three recently derived patient isolates were tested for their cytoadherence to purified receptors (CD36 and ICAM-1) as well as endothelial cells. Monoclonal antibodies against human CD36 and ICAM-1 were used to inhibit and reverse infected erythrocyte binding in static and flow-based adhesion assays.

**Results:**

Anti-ICAM-1 and CD36 monoclonal antibodies were able to inhibit and reverse *P. falciparum* binding of lab and recently adapted patient isolates in vitro. However, reversal of binding was incomplete and varied in its efficiency between parasite isolates.

**Conclusions:**

The results show that, as a proof of concept, disturbing existing ligand–receptor interactions is possible and could have potential therapeutic value for severe malaria. The variation seen in the degree of reversing existing binding with different parasite isolates and the incomplete nature of reversal, despite the use of high affinity inhibitors, suggest that anti-adhesion approaches as adjunct therapies for severe malaria may not be effective, and the focus may need to be on inhibitory approaches such as vaccines.

## Background

The understanding of the molecular mechanisms underpinning *Plasmodium falciparum*-infected red blood cell (pRBC) cytoadherence has provided a complex picture of pRBC. Adhesion to vascular endothelial cells (EC) is associated with the interaction of *Plasmodium falciparum* erythrocyte membrane protein 1 (PfEMP1) on the surface of red blood cells (RBC) and a range of host adhesion receptors expressed on microvascular EC. EC can express many different adhesion molecules on their surfaces that support adhesion of pRBC, including CD36, ICAM-1, EPCR, VCAM-1, E-selectin and PECAM-1 [1, 2]. The role and relative importance of these receptors in sequestration is still not clear, but receptor cooperation/synergism has been shown to enhance the binding [3, 4].

There is some evidence, although it is not supported by all studies, that interaction of PfEMP1 with ICAM-1 is involved in the pathogenesis of cerebral malaria [5] while adhesion to CD36 has been associated with uncomplicated malaria as well as non-cerebral severe malaria (see [6] for a review). More recent work has identified EPCR as being associated with severe malaria [2], including the possibility of structural conservation of the binding site on PfEMP1 that might support the development of a vaccine [7, 8]. Therefore, blocking and disrupting pRBC adhesion to host receptors could reduce the probability of developing severe malaria (SM).

Interfering with the parasite/host interaction by targeting PfEMP1 may reduce cytoadherence. PfEMP1 consists of multiple domains [Cysteine-rich Interdomain Region (CIDR) and the Duffy Binding-like (DBL) domains] and previous work has shown that an anti-CIDR antibody can block binding of pRBC to immobilized CD36 protein [9]. A specific PfEMP1 variant has been found expressed on pRBC associated with placenta malaria and subsequent studies based on the molecular characterization of this molecule (VAR2CSA) have derived cross-reactive antibodies able to inhibit pRBC binding to CSA [10]. The CSA binding site has been identified and studies are underway to test vaccine candidates based on this structure [11]. For malaria infections in non-pregnant hosts, the control of expression of *var* genes that produce PfEMP1 proteins is complex [12] and results in a high degree of variability of PfEMP1 expression due to antigenic variation. Thus, the use of antibodies based on PfEMP1 fragments other than VAR2CSA will not be simple and will require further work to define key binding signatures. Some progress has been made in this area with cross-reactive antibodies defined for PfEMP1 DC4 to ICAM-1 binding [13] and PfEMP1 DC8/DC13 binding to EPCR [7], including the structure of PfEMP1 showing dual binding specificity with these EC receptors [14].

The alternative to preventing interactions based on inhibition of PfEMP1 is by blocking endothelial receptors, which may solve the problem of variability of PfEMP1 in this system. It has been shown previously that some mAbs can inhibit the interaction of pRBC to specific receptors on EC. For example, mAb OKM5, which has as its epitope the immuno-dominant region at amino acids 139–184 of CD36, is able to block cytoadherence of pRBC to CD36 [15, 16]. Adhesion to ICAM-1 can be inhibited using several different mAbs against ICAM-1 [17, 18] such as mAb 15.2 against the L42 loop of domain 1 of ICAM-1. This approach appears to work across different PfEMP1 variants, including field isolates [13, 19], suggesting some conservation of the host binding site.

Several published experiments have addressed inhibition of adhesion of pRBC by mAb, focussing on the prevention of de-novo adhesion [20], but there have been very few studies looking at the potential to reverse existing pRBC cytoadherence [21]. Reversing pRBC sequestration has been considered as an attractive contributing strategy for the management of SM [22], as an adjunct to standard anti-parasite treatment. The rationale for reversing sequestration was based on the beneficial effects of administration of anti-malaria immunoglobulins from adults with malaria to children with mild malaria in Thailand [23]. The results suggested that the antibodies inhibited cytoadherence to C32 melanoma cells and rosette formation. This may be a natural preventive mechanism against the severity of *P. falciparum* infection in the infected host, and the effect has been replicated in animal models [24], and in in vitro adhesion studies [21, 25]. In a squirrel monkey model of malaria [24], the administration of hyperimmune serum was rapidly followed by the swift appearance (30 min after injection) of previously adherent infected erythrocytes in the peripheral circulation and an impressive recovery from sickness. In vitro, adhesion of Thai isolates to C32 melanoma cells was reversed with Thai immune sera [21], and the adhesion of isolates from Malawi was reversed with a pool of local immune sera [25]. Adhesion to individual receptors can be reversed with monoclonal antibodies [26–29] or ligand peptide segments [30, 31]. Recently investigators have used a modified heparin compound, sevuparin [32], to reverse adhesion, although the mechanism of action is not clear.

While the reversal of cytoadherence by human serum containing relevant antibodies, both in vitro and in vivo, suggests that a pool of high-titre malarial antibodies, shown to have reactivity with the surface of infected erythrocytes [25] could reverse adhesion in vivo, a double blind, placebo-controlled administration of the antibodies as an adjunct to quinine (the best available anti-malarial at the time) had no measurable observed effect on adhesion, and did not affect patient recovery [22]. The inherent difficulty with this approach is, at least in part (but see also [33]), the variable nature of PfEMP1 and, therefore, the complexity of the antibody pool to interfere with cytoadherence. As the host-parasite interaction has (at least) two components, another approach would be to block the host receptor, although this could have potentially serious adverse effects if the target overlapped with a critical host function. Not enough is known about the details of the binding sites for all the host receptors but available data show the pRBC binding site on ICAM-1 has discrete elements from the LFA-1 binding site [17], although the EPCR binding site uses the same region as that involved in the conversion of protein C to its activated form [2, 34, 35]. This study investigated whether antibodies to individual host receptors could reverse existing pRBC adhesion to endothelial cells under physiological conditions, using the common adhesion receptors ICAM-1 and CD36, both as purified proteins and in cellular context.

## Methods

### Parasite culture

ItG [36] and C24 [37] laboratory parasite lines, which are well characterized for their binding to ICAM-1 and CD36 respectively [38], were cultured under standard conditions in RPMI 1640 medium supplemented with 37.5 mM HEPES, 11 mM d-glucose, 0.2% NaHCO_3_, 25 μg/ml gentamycin sulfate, 2 mM l-glutamine and 10% pooled human serum at pH 7.2 in a gas mixture comprising 96% nitrogen, 3% carbon dioxide, and 1% oxygen. The culture-adapted ICAM-1-selected [19] parasite lines GL6, P069 and 8146 [19] were also investigated in reversal assays.

### Endothelial cell culture

Human umbilical vein vascular endothelial cells (HUVEC) and human dermal microvascular endothelial cells (HDMEC) obtained from Promocell were cultured as per manufacturer’s procedures. Cells at passage 4–6 were used for all experiments. Prior to use, cells were stimulated by addition of 1 ng/ml TNF for 18 h to allow enhanced ICAM-1 expression on the surface of the EC.

### Plasmagel trophozoite enrichment

The parasite culture was centrifuged at 500*g* for 5 min and the pellet resuspended in a ratio of 2 volumes pellet to 3 volumes RPMI-based growth media without human serum (incomplete medium) and 5 volumes Plasmion (Fresenius Kabi), and allowed to settle for 20–30 min at 37 °C. Trophozoite stage pRBC in the top layer were then washed three times in incomplete medium and the parasitaemia assessed by Giemsa-stained smear.

### Selection of pRBC on ICAM-1 purified protein

To increase the homogeneity of the ItG parasite population, which expresses a PfEMP-1 variant with high affinity for ICAM-1, the population was subjected to selection on ICAM-1 protein. 2.5 µg of ICAM-1 protein [39] was coated on 50 µl of protein A Dynabeads (Invitrogen) in 400 µl of 1% bovine serum albumin (BSA) in PBS and incubated for 1 h at room temperature with gentle rotation (15 rpm). Dynabeads were washed gently in 1% BSA/PBS using a magnetic stand. 50 µl of ItG parasite culture synchronized and enriched using Plasmagel enrichment were incubated with the coated beads in 400 µl BSA/PBS for 45 min at room temperature by gentle rotation. Unbound pRBC were removed and the bound pRBC washed three times with BSA/PBS using the magnetic stand. Beads were resuspended in 5 ml of complete RPMI media and transferred to a culture flask with the addition of 50 µl of washed red blood cells.

### Inhibition and reversal assays with purified receptor under static conditions

Bacteriological petri-dishes (6 cm) coated with 50 μg/ml CD36 and 50 μg/ml ICAM-1 protein spots [18] were pre-incubated with 1.5 ml of binding buffer (RPMI 1640 medium with 25 mM HEPES, 11 mM d-glucose, 2 mM l-glutamine, pH 7.2) with or without 5 µg/ml αICAM-1 antibody (clone 15.2; Santa Cruz) or 10 µg/ml αCD36 antibody (clone FA6-152; abcam), at 37 °C for 30 min, before proceeding with the adhesion assay. The solutions were aspirated, a parasite suspension of 3% parasitaemia and 1% haematocrit in binding buffer was added and incubated for 1 h at 37 °C rotating every 10 min. The petri-dish was washed 3–5 times with binding buffer, fixed using 1% glutaraldehyde for at least 1 h and then stained with 5% Giemsa for 30 min.

Reversal assays were carried out in a similar fashion, except that pRBC binding was performed without antibody. After the 3–5 washes in binding buffer, the dishes were incubated for a further 1 h with either binding buffer alone, or with the addition of 5 µg/ml αICAM-1 antibody or 10 µg/ml αCD36 antibody, with gentle mixing every 10 min. Dishes were washed with 4 × 2 ml binding buffer, fixed in 1% glutaraldehyde and stained with Giemsa. All experiments were carried out with duplicate dishes each containing triplicate spots.

### Reversal assays with purified receptor under flow conditions

Flow assays were carried out on microslides coated with ICAM-1 or CD36 at 50 µg/ml. Slides were prepared and assays carried out as described previously [38]. pRBC at 3% parasitaemia and 1% haematocrit in binding buffer were flowed through the microslide for 5 min to allow for pRBC adhesion. Flow was continuous throughout the experiment at 0.05 Pa shear stress. After 5 min, the fluid was switched to binding buffer for 2 min to remove unbound pRBC and clear flow lines of pRBC. Timing was started with flowing through binding buffer containing no antibody (control), 5 µg/ml αICAM-1 or 10 µg/ml αCD36 antibodies. The number of bound pRBC in six fields along the slide was counted at 0, 5, 10, 15 and 20 min time points.

### Inhibition and reversal assays with endothelial cells under static conditions

TNF-activated HUVEC or HDMEC were seeded on to coverslips (Nunc) and static cell assays carried out as previously described [38]. For the inhibition assay, the cells were pre-incubated with 1.5 ml of binding buffer with or without antibody (5 µg/ml αICAM-1 or 10 µg/ml αCD36), at 37 °C for 30 min, before proceeding with the adhesion assay. pRBC (3% parasitaemia and 1% haematocrit) were allowed to bind and, following two dip washes, coverslips were placed in a gravity wash for 30 min. The coverslips were transferred to a second gravity wash for 10 min, fixed in 1% glutaraldehyde and stained with 5% Giemsa.

For the reversal assay, after pRBC had been allowed to bind without any antibodies, and following two dip washes coverslips were placed cell-side up into a well containing binding buffer without (control) and with 5 µg/ml αICAM-1 or 10 µg/ml αCD36 mAb. As above, after 30 min the coverslips were transferred to a second gravity wash for 10 min, fixed in 1% glutaraldehyde and stained with 5% Giemsa.

Following Giemsa staining, coverslips were dried and mounted on slides using DPX mountant (Sigma). Levels of adhesion were quantified by microscopy under 200× magnification. The number of adherent pRBC per mm^2^ was calculated. All cell assays were carried out in triplicate.

### Reversal assays with endothelial cells under flow conditions

Reversal of adhesion to HUVEC or HDMEC under flow conditions was carried out using TNF stimulated HUVEC or HDMEC grown overnight on chamber slides, and assays were carried out as previously described [38]. pRBC at 3% parasitaemia and 1% haematocrit were flowed through the slide for 5 min to allow for pRBC adhesion. Flow was continuous throughout the experiment at 0.05 Pa shear stress. After 5 min pRBC suspension flow, the fluid was switched to binding buffer to remove unbound pRBC. Timing was started with flowing through binding buffer containing no antibody (control), 5 µg/ml αICAM-1 or 10 µg/ml αCD36 mAb. The number of bound pRBC in six fields along the slide was counted at 0, 5, 10, 15 and 20 min time points.

### Data analysis

For each experiment, the number of bound pRBC was calculated using Image-Pro Plus software (Media Cybernetics) and expressed as the mean bound pRBC/mm^2^ ± standard deviation. Statistically significance compared to the control (no Ab) was determined by t test.

## Results

### Effect of mAb inhibiting cytoadherence on purified protein

**Fig. 1:**
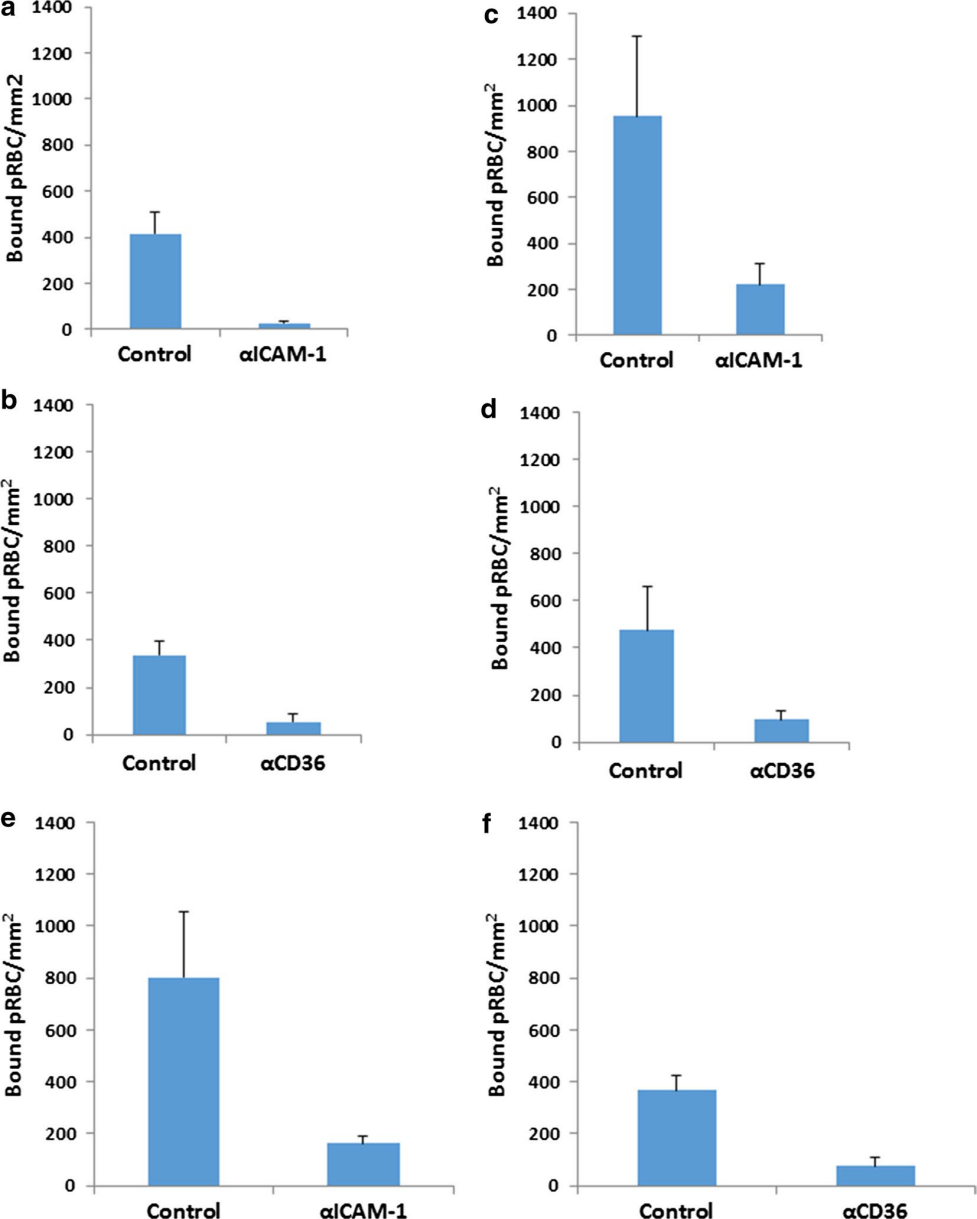
Inhibition of ItG binding to ICAM-1 under static (**a**) and flow (**c**) conditions. Binding in the absence of mAb (control) and after pre-incubation with αICAM-1 mAb at 5 µg/ml for 1 h. **b** Inhibition of C24 binding to CD36 under static and **d** flow conditions. Binding in the absence of mAb (control) and after pre-incubation with αCD36 mAb at 10 µg/ml for 1 h. Reversal of ItG binding to ICAM-1 (**e**) and C24 binding to CD36 (**f**) under static conditions. pRBC were allowed to bind to the protein for 1 h and binding was determined after subsequent incubation with 5 µg/ml αICAM-1 mAb (**e**) or with 10 µg/ml αCD36 mAb (**f**) for 1 h. Results expressed as mean bound pRBC/mm^2^ ± standard deviation. Control: without antibody

**Fig. 2:**
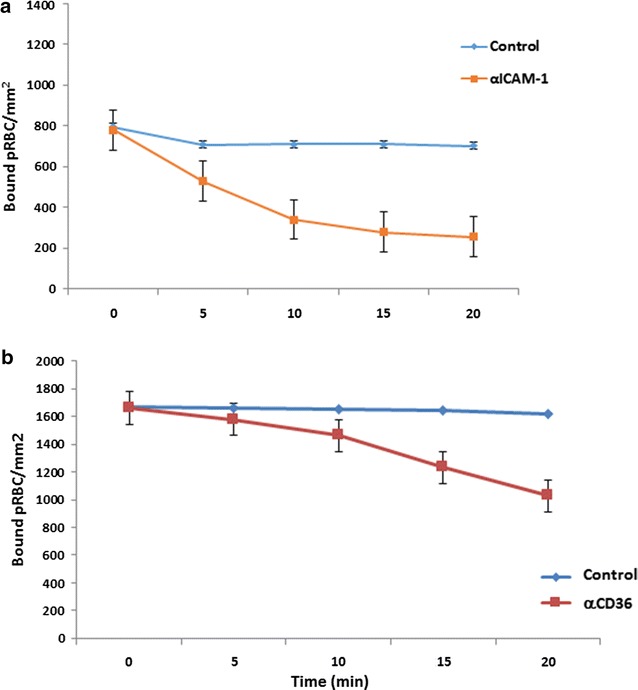
Reversal of ItG binding to ICAM-1 (**a**) and C24 binding to CD36 (**b**) under flow conditions. pRBC were allowed to bind to the protein for 5 min and binding was observed after flowing through binding buffer with αICAM-1 mAb at 5 µg/ml for 20 min (**a**) and αCD36 mAb at 10 µg/ml for 20 min (**b**). pRBC bound were determined every 5 min and expressed as bound pRBC/mm^2^. Control: without antibody

*Plasmodium falciparum* lab isolates ItG and C24 were selected for static and flow inhibition studies. Results show that ItG binding with αICAM-1 mAb (Fig. 1a, c) and C24 binding with αCD36 mAb (Fig. 1b, d) were significantly inhibited (*P* < 0.001) at levels of more than 80% inhibition under static and flow conditions, showing that binding of ItG and C24 was largely determined by ICAM-1 and CD36 respectively. Based on these results, we studied these two antibodies for their ability to reverse adhesion of already bound pRBC on protein (ICAM-1 and CD36). αICAM-1 mAb reverses binding of ItG and αCD36 mAb reverses binding of C24 under static conditions up to 80% (Fig. 1e, f, respectively), and under flow conditions approximately 60 and 35% (Fig. 2a, b, respectively), at 20 min timepoint.

### Effect of mAb inhibiting cytoadherence on endothelial cells

**Fig. 3:**
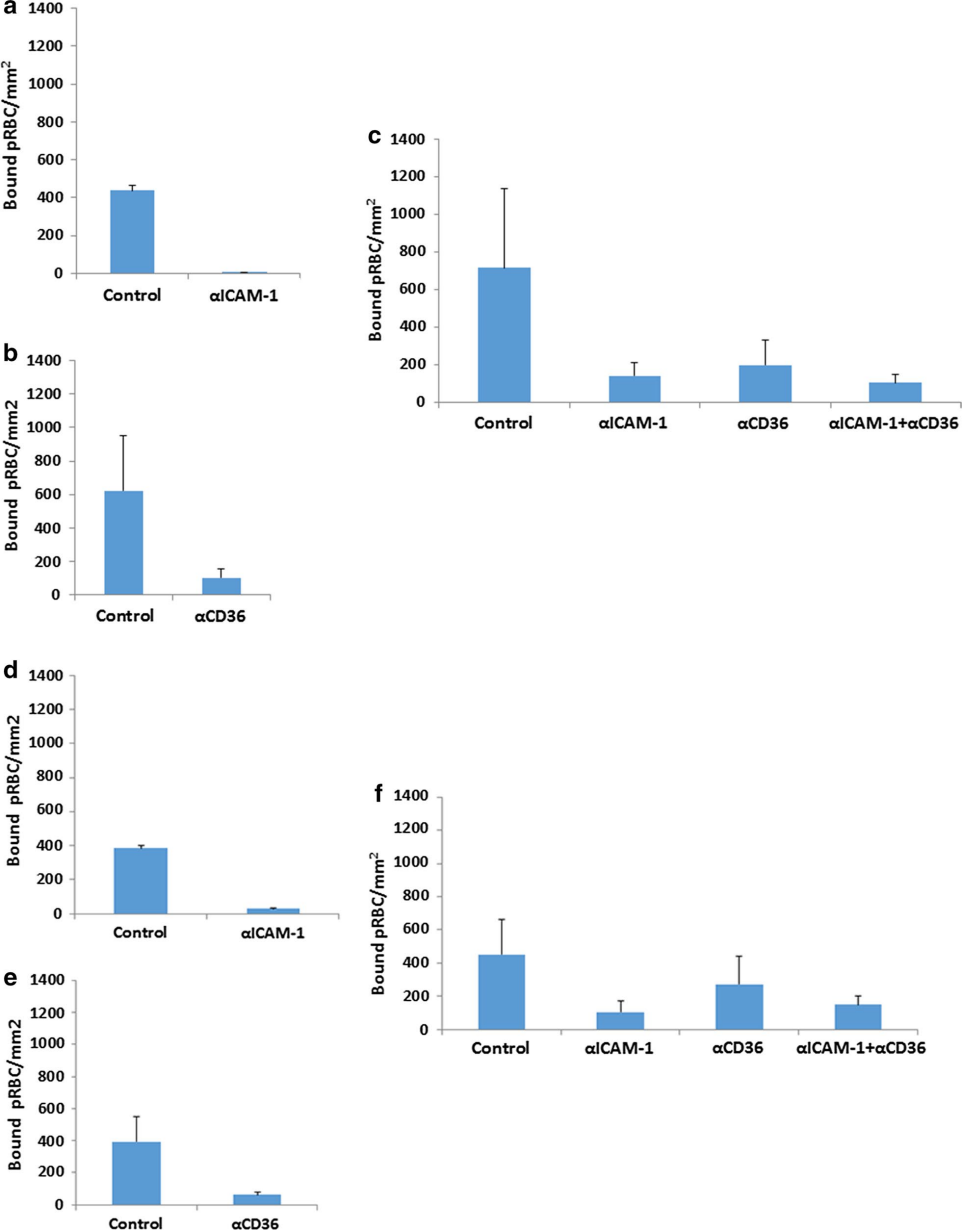
**a** Inhibition of ItG binding to TNF-stimulated HUVEC under static conditions observed after pre-incubation with 5 µg/ml αICAM-1 mAb for 1 h. **b** Inhibition of C24 to TNF-stimulated HDMEC under static conditions observed after incubation with 10 µg/ml αCD36 mAb for 1 h. **c** Inhibition of ItG binding to TNF-stimulated HDMEC under static conditions observed after incubation with 5 µg/ml αICAM-1 and 10 µg/ml αCD36 mAbs independently and the combination of both mAbs, for 1 h. **d** Reversal of ItG binding to TNF-stimulated HUVEC under static conditions. pRBC were allowed to bind to the cells for 1 h and binding was observed after incubation with 5 µg/ml αICAM-1 mAb for 1 h. **e** Reversal of C24 binding to TNF stimulated HDMEC under static conditions observed after incubation with 10 µg/ml αCD36 mAb for 1 h. **f** Reversal of ItG binding to TNF-stimulated HDMEC under static conditions observed after incubation with 5 µg/ml αICAM-1 mAb and 10 µg/ml αCD36 mAb or the combination of these mAbs, for 1 h. Results expressed as mean bound pRBC/mm^2^ ± standard deviation. Control, without antibody

Figure 3 shows that adhesion of ItG, which has strong binding to ICAM-1 and some to CD36, to HUVEC and HDMEC under static conditions was inhibited significantly using αICAM-1, αCD36 and both αICAM-1 and αCD36 mAbs combined (P < 0.01) (Fig. 3a, c) in comparison with ItG control (without antibody). Meanwhile C24 pRBC, which binds to CD36 but not ICAM-1, shows a reduction of binding after αCD36 mAb exposure (Fig. 3b). Results show that either αICAM-1 mAb individually or in combination with the αCD36 mAb, produce the same level of pRBC inhibition for ItG adhesion to HDMEC. This supports earlier findings of cooperative binding-interactions of some pRBC, including ItG, dependent on ICAM-1 in mediating efficient adhesion pRBC to the host endothelial cell [38], despite having some affinity for CD36 as well.

### Effect of mAb reversing cytoadherence on endothelial cells

Based on the inhibition of binding to EC seen with the mAbs to CD36 and ICAM-1, reversal of adhesion of pRBC to HUVEC and HDMEC was investigated. To examine this effect static EC binding assays were conducted and then treated with the relevant mAb(s) for another 1 h at 37 C. Figure 3d shows that the binding of ItG to HUVEC was reduced more than 90% after treatment with 5 µg/ml αICAM1 mAb and the binding of C24 to HDMEC was reduced 85% after treatment with 10 µg/ml αCD36 mAb in comparison with the control (Fig. 3e) (both P < 0.05). The reduction of ItG binding to HDMEC by αICAM1 mAb was significant at ~75% (P < 0.05), but only 40%, and not significant, by αCD36 mAb in comparison with the control. The combination of αICAM1 and αCD36 mAbs reversed the binding 66%, similar to αICAM1 mAb on its own (Fig. 3f).

**Fig. 4:**
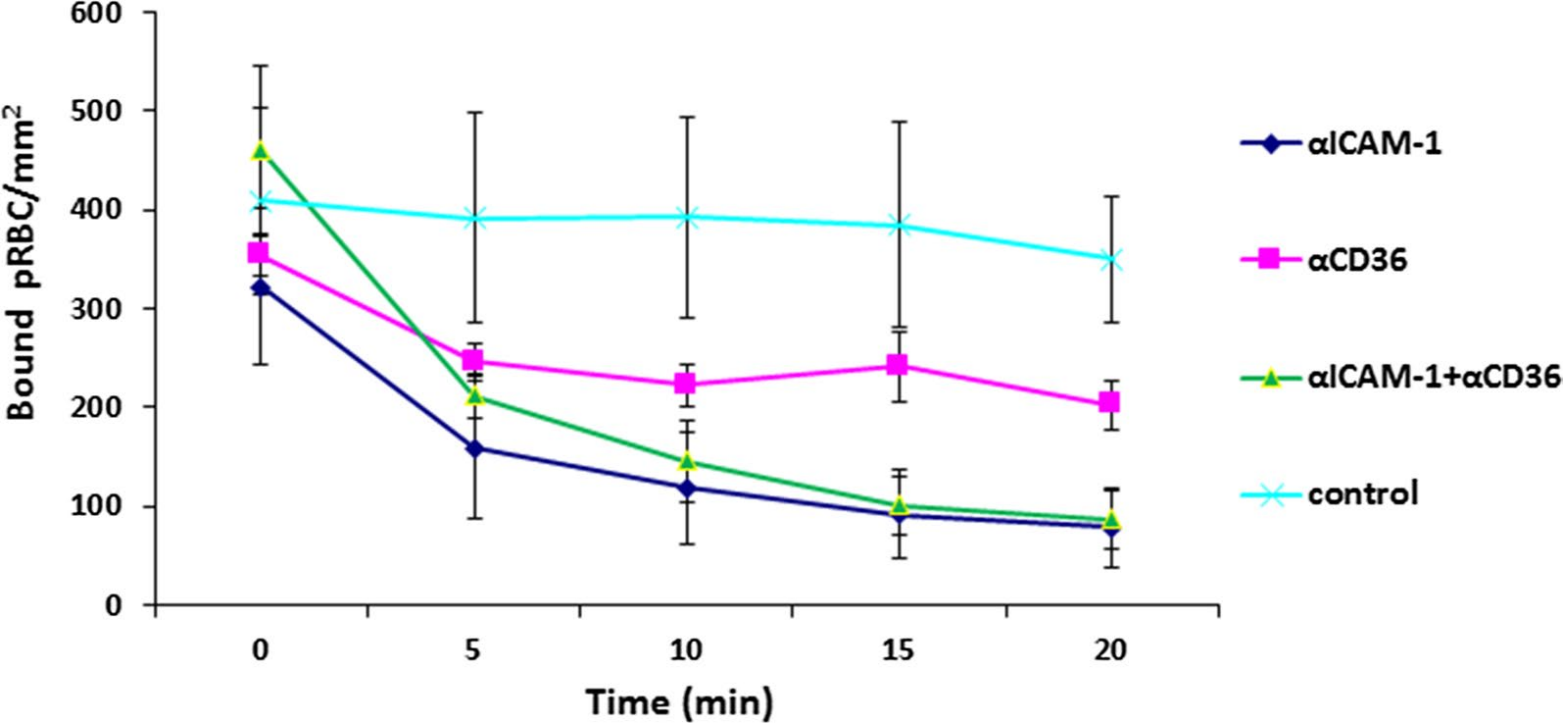
Reversal of ItG binding on TNF-stimulated HDMEC under flow conditions. pRBC were allowed to bind to the EC under flow for 5 min, washed for 2 min and then a solution of BB without mAb (control) or with 5 µg/ml αICAM-1 mAb, 10 µg/ml αCD36 mAb and a combination of both mAbs were used to reverse binding. The number of bound pRBC was counted at 0, 5, 10, 15 and 20 min. Results expressed as mean bound pRBC/mm^2^ ± standard deviation

With the successful reversal of the sequestered pRBC on static cell based assays, endothelial cell flow based assays were performed following 20 min exposure to αICAM-1 and αCD36 mAbs, with significant reduction, and similar to the static assay, of ItG binding (Fig. 4) to HDMEC seen in comparison with the control.

### Effect of mAb reversing adhesion of patient isolates to endothelial cells

**Fig. 5:**
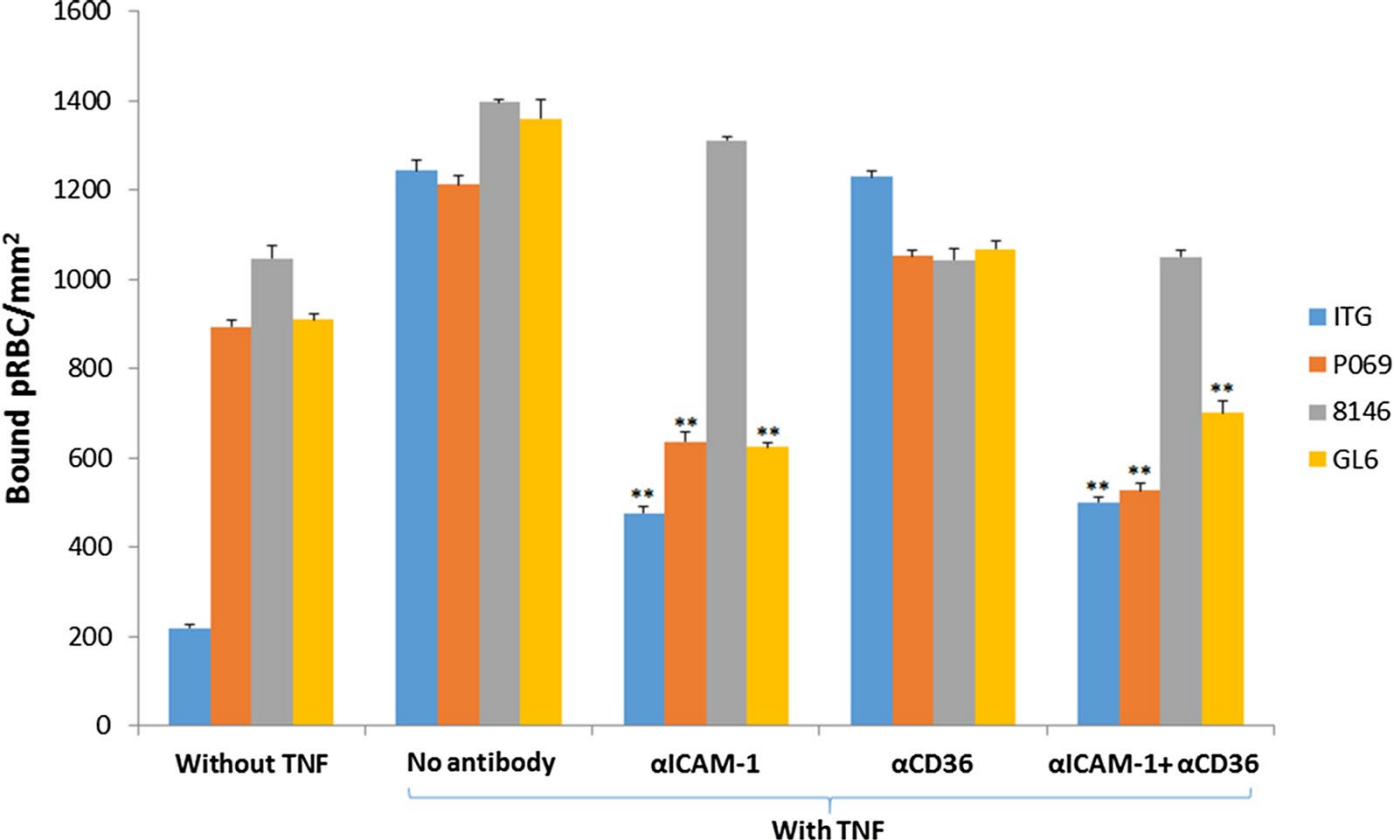
Reversal of binding of the culture-adapted clinical isolates, P069, 8146 and GL6, on unstimulated and TNF-stimulated HDMEC under static conditions. pRBC, including ItG, were allowed to bind for 1 h to the EC, washed and subsequently incubated in BB without mAb (control) or with αICAM-1 mAb at 5 µg/ml and αCD36 mAb at 10 µg/ml or the combination of these mAbs for 1 h. Results expressed as bound pRBC/mm^2^ ± standard deviation. **P < 0.01 (compared with group “with TNF”). Binding is also shown to unstimulated (“without TNF”) HDMEC

The effects of αICAM-1 and αCD36 mAbs on adhesion to TNF stimulated HDMEC were further investigated using culture-adapted patient isolates (GL6, P069 and 8146), which have been characterized for their binding properties to ICAM-1 [19]. The results for the static assay show that αICAM-1 mAb alone is able to reverse P069 and GL6 binding, but not 8146 binding. Meanwhile, αCD36 mAb alone shows only slight decreases (15–25%) with all isolates’ binding to TNF-stimulated HDMEC. However, when both mAbs (αICAM-1 + αCD36) are combined, a similar level of binding reduction was produced compared to either αICAM-1 or αCD36 mAb alone, depending on which one had the most effect as a single mAb treatment (Fig. 5).

**Fig. 6:**
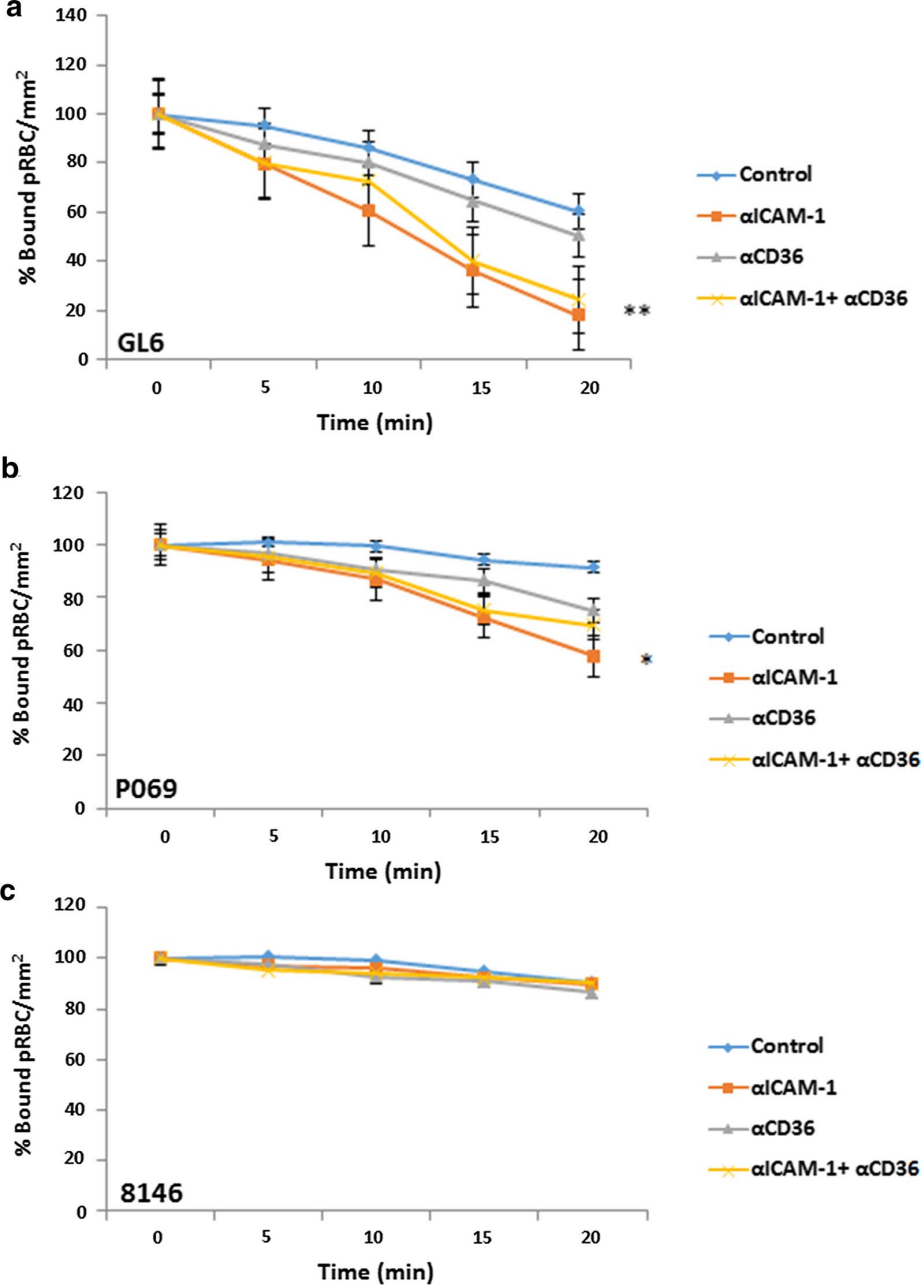
Reversal of GL6 (**a**), P069 (**b**) and 8146 (**c**) binding on TNF-stimulated HDMEC under flow conditions. pRBC were allowed to bind to the cells under flow for 5 min, washed for 2 min and then a solution of BB without mAb (control) or with 5 µg/ml αICAM-1 mAb, 10 µg/ml αCD36 mAb or a combination of both was used for to reverse binding. The number of bound pRBC was counted at 5, 10, 15 and 20 min and expressed as percentage (%) bound pRBC/mm^2^ compared to 0 min. *P < 0.05 (compared to 0 min); **P < 0.01 (compared to 0 min)

Under flow conditions, the αICAM-1 mAb, showed loss of binding over 20 min for GL6 (70%) and PO69 (37%). However, αCD36 reversed binding by only 17–18%, while 8146 binding was not reversed by either mAb (Fig. 6).

## Discussion

Cytoadherence is thought to be a major virulence factor involved in the pathogenesis of severe malaria. It has been observed that most malaria deaths in children occur in the first 24–48 h of hospitalization, despite effective anti-malarial regimes (quinine or artemisinin) given immediately once admitted [40–42]. Hence, reversing the sequestration of pRBC could be an important approach for the development of adjunctive therapy for severe disease. The ability of anti-adhesion mediators to not only inhibit, but also to reverse adhesion has important therapeutic implications. Hughes et al. have shown cytoadherence of pRBC to EC can continue for several hours after administration of anti-malarial drug treatment [43] despite the pRBC being dead. The continuing sequestered parasite load has the potential to contribute to disease severity by impeding blood flow and causing endothelial damage, as well as enhancing local inflammatory responses [44].

In this study, the ability of mAbs to inhibit and reverse the cytoadherence of pRBC on purified protein (ICAM-1 and CD36) and endothelial cells HUVEC and HDMEC was assessed. The data demonstrate that αICAM-1 (15.2) and αCD36 (FA6-152) mAbs not only highly significantly inhibit, but also significantly reverse *P. falciparum* pRBC cytoadherence on protein and EC in vitro, although not all isolates showed reversal (e.g. 8146).

Complex interactions of pRBC to the endothelial receptors ICAM-1 and CD36 have been shown to occur through DBLβ and CIDRα domains of PfEMP1 [45, 46] via the N-terminal (domain 1) of ICAM-1 [17] and the phosphorylated ectodomain of CD36 respectively [47]. The binding of pRBC to ICAM-1 has some overlap with that between ICAM-1 and human rhinovirus and LFA-1 [17], using a similar region of the host receptor for the pathogen and the critical host pathway to interact, but having variation in the contact residues used. The latter is particularly important as the discrimination by mAbs between blocking LFA-1 and pRBC binding could be important in the design of potential therapies. Blocking ICAM-1-dependent interactions has been demonstrated by the ability of anti-LFA-1 mAbs to inhibit and reverse sequestered leucocytes from endothelium (binding to ICAM-1) in murine models, and shows the potential of these types of reagents to inhibit and reverse binding, in a way that could be applicable to pRBC cytoadherence. Therefore, by postulating the same approach to inhibit and reverse pRBC binding to ICAM-1, the use of mAbs against the surface protein PfEMP1 would seem to be warranted. Unfortunately, this is not simple due to the high level of variation seen in *var* genes, which encode PfEMP1, although some progress has been made in identifying conserved PfEMP1 structures responsible for EPCR binding [7], which could be used as the basis for vaccines. In addition, because not all PfEMP1 variants cause SM, finding the appropriate *var* sequences to use for a PfEMP1-based vaccine could be difficult, despite recent advances in characterizing conserved regions in this family [48]. Therefore, an approach to inhibit pRBC adhesion based on the host receptor was considered here.

Anti-ICAM-1 mAb15.2 was chosen because it has been used previously and shown to be able to inhibit binding of pRBC to ICAM-1 through interaction at loop 42 domain of ICAM-1, seen in a range of upsA and upsB PfEMP1 variants [13, 18]. MAbFA6-152 was chosen based on its ability to inhibit pRBC adhesion on CD36, recognizing an epitopes within the region 155–183 of CD36 used for pRBC binding [49]. The study was initially carried out on two laboratory-adapted *P. falciparum* lines, ItG and C24. ItG is a *P. falciparum* line expressing a PfEMP1 encoded by ITvar16, which binds to ICAM-1 strongly, but less to CD36, while the C24 line has a PfEMP1 that is encoded by ITvar24 and binds to CD36 stronger than ItG, but does not bind to ICAM-1.

Both mAbs significantly inhibit pRBC adhesion to ICAM-1 and CD36 under static and flow conditions, consistent with other studies, but also show the ability to reverse existing adhesion, with αICAM-1 mAb more effective under flow conditions than αCD36 mAb (Figs. 1, 2). Following successful reversal and inhibition of binding to purified protein with both parasite strains, assays were carried out using TNF stimulated primary human EC (HUVEC and HDMEC). Assays using ItG were conducted using both mAbs individually, and in-combination (for HDMEC) to observe any cooperative activity between these receptors. Cytoadherence under flow conditions is thought to involve multiple adhesion receptors acting cooperatively, as shown with CD36, ICAM-1 and P-selectin [3, 38, 50]. A model has been widely proposed where pRBC are captured from flow by one receptor, roll along EC before firm adhesion takes place, possibly being mediated by a different receptor. This may separate out roles for different receptor families, where a selectin (i.e. P-selectin) or Ig superfamily receptor (i.e. ICAM-1) may be required to capture PRBC from flow followed by firm adhesion to a different class of receptor i.e. CD36. Firm adhesion may also require or be enhanced by interactions with more than one receptor concurrently (i.e. ICAM-1 and CD36), and pathology may also require combinations of receptors (i.e. ICAM-1 and EPCR [51]).

Binding of C24 to HDMEC under static conditions was inhibited and reversed by ~85% by an αCD36 mAb (Fig. 3b, e). Binding of ItG was inhibited by both antibodies individually, and at a similar level when both antibodies were used in combination. This suggests that either receptor (ICAM-1 and CD36) is required for efficient binding for a population of pRBC (Fig. 3c), for ItG. The results from reversal experiments using ItG illustrated that αICAM-1 mAb and the combination (αICAM-1 + αCD36 mAbs) give similar efficacy, whereas αCD36 mAb alone is less effective, suggesting that ICAM-1 plays a more significant role for binding of this ItG line (Fig. 4). These results support observations by McCormick et al., where CD36 was found at lower densities than ICAM-1 on TNF-stimulated HDMEC [3], and suggest that an inhibitor based on a single adhesion component might be effective in reducing cytoadherence.

These characteristics were further explored by examining the ability of the mAbs to reverse recently culture-adapted patient isolates (GL6, P069 and 8146) in comparison with the laboratory parasite line (ItG) (Fig. 5). The results show that the αICAM-1 mAb was more effective at reversing binding than αCD36 mAb under flow and static conditions for GL6 and PO69, but at a lower level compared to ItG. In one case (8146) there was almost no reversal of adhesion, which was different from previously published results [19] and may indicate a mixed population due to PfEMP1 switching during in vitro culture. This suggests that the clinical isolates tested might be expressing PfEMP1 variants with possibly more extensive repertoires of host receptor binding and highlights a challenge of using anti-adhesion approaches for therapy due to the need to include inhibition of multiple receptors. Further work will be needed to identify whether reasonable coverage could be achieved in reversing cytoadherence using inhibitors based one or two specific receptors (i.e. ICAM-1 and EPCR).

Several previous studies have shown that antibodies can work through inhibiting the binding of pRBC to specific receptors but have not looked at their ability to reverse established cytoadherence, despite this being the likely situation encountered for an adjunct therapy. To reverse existing adhesion, it is necessary that the affinity of mAbs for the corresponding receptor is higher than that of the PfEMP1/host receptor interaction. Measurements of PfEMP1/receptor K_d_ are around 1–100 nM [7, 52] (the ITvar16 (ItG)/ICAM-1 K_d_ is 51.1 nM) and mouse monoclonal mAbs have K_d_ values in the low nM to sub-nM range so it might be expected that the equilibrium of mAb and pRBC ligand for the receptor would allow the antibody to compete for the functional binding site, freeing the bound pRBC into the microvasculature [33]. The type of antibody needs to be chosen carefully, as it should not interfere with the physiological role of the specific endothelial receptor. However, the multivalent interaction of pRBC with EC could compete well with mAbs (or inhibitors) in solution, therefore reversing adhesion in vivo could be a challenge, which supports the relatively slow loss of pRBC from the EC under flow seen here for some parasite isolates.

The data demonstrate that mAbs can both inhibit and reverse binding under static and flow conditions, albeit with only partial reversal seen with patient isolates. Whilst this gives some support for the concept of an anti-adhesion therapy, which could minimize the severe complications of falciparum malaria, there are limitations on the design of these inhibitors, in particular the need for high affinity to compete with PfEMP1/host interactions. With cerebral malaria patients, the intravenous introduction of adhesion-blocking substances could not only lead to reversal of sequestration, but may also prevent the onset of brain swelling in cerebral malaria [53] through reducing pRBC-induced endothelial activation.

The results suggest that SM might be managed through anti-adhesion therapy, but it needs to be borne in mind that as well as being receptors for parasite adhesion, ICAM-1 and CD36 play major roles in leukocyte trafficking and normal human immune system responses. It has been shown that in blocking the receptor protein for the pathogen, this might inhibit the normal function of the protein and possibly affect the host [54]. Therefore, while these experiments might show the concept of anti-adhesion development based on receptor competition, further work should be considered for the development of anti-adhesion therapies based on specificity for the pRBC interaction, high affinity and, for practical reasons, cost of goods. Consideration about the effect of releasing many rigid pRBC into the spleen also needs to be given. Taken together, these represent barriers to the development of anti-adhesion adjunct therapies, although interpretation of the results obtain in this paper needs some caution as only at a limited number of parasite lines were examined and only for the receptors ICAM-1 and CD36. Also, anti-adhesion strategies may be able to directly reduce pathology, for example by removing EPCR-binding infected erythrocytes from the activated protein C generating site on EPCR.

While the ability to reverse existing pRBC cytoadherence to EC has been demonstrated in this paper, the results suggest that looking at other approaches such as those based on mitigating the effects of cytoadherence on the vasculature, rather than the adhesion itself or the induction of cross-reactive immunity to binding by vaccination, may be more viable approaches. Further work on a more extensive panel of parasite lines and receptor combinations is required, but the integration of reversal assays into anti-adhesion product development is warranted.
